# Contribution of protein intake and its interaction with physical activity to transitions between disability states and to death in very old adults: the Newcastle 85+ Study

**DOI:** 10.1007/s00394-019-02041-1

**Published:** 2019-07-10

**Authors:** Nuno Mendonça, Andrew Kingston, Antoneta Granic, Tom R. Hill, John C. Mathers, Carol Jagger

**Affiliations:** 1 Newcastle University Institute for Ageing, Newcastle University, Newcastle upon Tyne, NE2 4AX UK; 2 Human Nutrition Research Centre, Newcastle University, Newcastle upon Tyne, NE2 4HH UK; 3 Institute of Health and Society, Newcastle University, Newcastle upon Tyne, NE4 5PL UK; 4 AGE Research Group, Institute of Neuroscience, Newcastle University, Newcastle upon Tyne, NE2 4HH UK; 5 NIHR Newcastle Biomedical Research Centre in Ageing and Chronic Disease, Newcastle University and Newcastle upon Tyne NHS Foundation Trust, Newcastle upon Tyne, NE4 5PL UK; 6 Institute of Cellular Medicine, Newcastle University, Newcastle upon Tyne, NE2 4HH UK

**Keywords:** Malnutrition, ‘Aged, 80 and over’, Multi-state, PROMISS, Disability, Protein, Physical activity, Transitions

## Abstract

**Introduction:**

Growth in the number of very old (≥ 85 years) adults will likely lead to increased prevalence of disability. Our aim was to determine the contribution of protein intake, and the interaction between protein intake and physical activity (PA), to the transition between disability states and to death in the very old using the Newcastle 85+ Study.

**Methods:**

The analytic sample comprised of 717 older adults aged 85 years at baseline and living in the community. Protein intake was estimated with 2 × 24-h multiple pass recalls (24 h-MPR) at baseline. Disability was measured as difficulty performing 17 activities of daily living (ADL) at baseline, at 18, 36, and 60 months, and defined as having difficulties in one or more ADL. The contribution of protein intake [g/kg adjusted body weight/day (g/kg aBW/d)] to transition probabilities to and from disability, and to death over 5 years was examined by multi-state models adjusted for key health covariates.

**Results:**

Participants were expected to spend 0.8 years (95% CI 0.6–1.0) disability-free and 2.8 years (95% CI 2.6–2.9) with disability between the ages 85 and 90 years. One unit increase in protein intake (g/kg aBW/d) halved the likelihood of incident disability (HR 0.44, 95% CI 0.24–0.83) but not for other transitions. Similar reductions in disability incidence were also found in individuals with protein intake ≥ 0.8 (HR 0.50, 95% CI 0.31–0.80) and ≥ 1 g/kg aBW/d (HR 0.49, 95% CI 0.33–0.73). Participants with high PA and protein intake ≥ 1 g/kg aBW/d were less likely to transition from disability-free to disability than those within the same PA level but with protein intake < 1 g/kg aBW/d (HR 0.45, 95% CI 0.28–0.72).

**Conclusion:**

Higher protein intake, especially in combination with higher physical activity, may delay the incidence of disability in very old adults.

**Electronic supplementary material:**

The online version of this article (10.1007/s00394-019-02041-1) contains supplementary material, which is available to authorized users.

## Introduction

The number of very old adults (≥ 85 years) is increasing to the point where it is now the fastest growing age group in most western societies [1]. Ageing is frequently accompanied by functional decline which leads to disability. Although it has been questioned whether population ageing necessarily leads to an increase in disability prevalence [2], past trends for the UK indicate that the proportion of the very old living in the community with medium and high dependence increased from 66 and 26% in 1991 to 86% and 48% in 2011, respectively [3]. Strategies to delay the onset of disability are, therefore, of high public health interest, and achieving adequate protein intake has been proposed as one such strategy as it may decrease the loss rate of muscle strength, bone health, and physical function [4, 5]. On average, older adults have lower protein intakes than younger individuals largely due to multi-morbidity, reduced physical activity, anorexia of ageing, and loss of independence [6–9]. This may be coupled with disease-related catabolism, inflammation, and anabolic resistance which would increase protein requirements leading to a further imbalance between protein intake and protein requirements [8, 10, 11].

We have shown that protein intake < 1 g/adjusted body weight/day (aBW/d) negatively affected muscle strength and physical performance in the very old [12]. We have also identified four disability trajectories in very old adults and demonstrated that higher protein intake, especially ≥ 1 g/kg aBW/d, was associated with a greater likelihood of remaining disability-free [13]. In the present study, our aim is to investigate the relationship between protein intake and disability further using multi-state models to assess the effect of protein intake on disability incidence, recovery from disability, and transition from disability to death. In addition, we explore the role of physical activity (PA) in the protein–disability relationship, which was not possible with the previous model [13]. We investigate these relationships in the Newcastle 85+ Study, a large and socio-demographically representative cohort of very old adults, as individuals age between 85 and 90 years.

## Methods

### Newcastle 85+ Study

The Newcastle 85+ Study is a longitudinal population-based study that approached all people turning 85 in 2006/2007 (born in 1921) in Newcastle and North Tyneside, UK. At baseline, the analytic sample consisted of 717 very old adults living in the community, with complete protein intake assessment, with body mass index (BMI) calculated and disability count. Full details of the Newcastle 85+ Study have been published [14]. A flowchart of the recruitment and retention profile of the Newcastle 85+ Study is presented in Fig. S1. The Newcastle 85+ Study was approved by the Newcastle and North Tyneside Local Research Ethics Committee 1 and in accordance with the Declaration of Helsinki. Signed consent was obtained from each participant and a signed consultee approval was obtained whenever the patient lacked capacity.

### Protein intake

Dietary intake was assessed by two 24 h multiple pass recall (24 h-MPR) on non-consecutive occasions administered by a research nurse in the participant’s usual place of residence at baseline. Energy and protein intake were estimated using the McCance and Widdowson’s sixth edition food composition tables [15]. Details of the dietary intake assessment can be found elsewhere [16]. Twenty-six percent of the participants were identified as possible misreporters (most being underreporters) using an energy intake:basal metabolic rate (EI:BMR) cut-off of 1.05–2.00 [16]; these have not been excluded from the main analysis because of the uncertainty surrounding this estimate and the small differences in population nutritional intakes between excluding and not excluding misreporters [16].

### Physical activity

A purposely designed physical activity questionnaire (PAQ) categorised participants into low (scores 0–1), medium (scores 2–6), and high (scores 7–18) PA according to the frequency and intensity of PA per week at baseline [17]. The PAQ includes questions on how frequently [≥ 3 times per week (score of 3), 1–2 times per week (score of 2), 1–3 times per month (score of 1), and hardly ever (score of 0)] the participants engaged in mildly energetic (e.g., light gardening and light housework), moderately energetic (e.g., moderate gardening, walking at moderate pace, and heavy housework), and highly energetic activities (e.g. heavy gardening, swimming, and cycling) (the PA questionnaire can be found at http://research.ncl.ac.uk/85plus/). The resulting total PA score was calculated as 3 × highly energetic activities + 2 × moderately energetic activities + mildly energetic activities. The PAQ was validated against a triaxial accelerometer (GENEA, Unilever, UK) continuously worn for 5–7 days on the right wrist [17]. Briefly, 484 participants in wave 2 of the Newcastle 85+ Study completed the PAQ and 337 wore a triaxial accelerometer (GENEA, Unilever, UK) for 5–7 days on the right wrist. Self-reported PA was significantly associated with objective measures of PA and these were significantly different when low, moderate, and high self-reported PA categories were compared (all *p* < 0.001).

### Disability

The disability score was calculated at baseline and at 18, 36, and at 60 months of follow-up by summing the self-reported difficulty performing 17 activities of daily living (ADLs), such as getting in and out of bed, cutting own toenails and walking 400 yards (366 m) (Fig. S2). Each participant scored 1 for each activity that could not be performed or was performed with any difficulty and 0 without difficulty. We have shown that self-reported difficulty in performing mobility ADLs was strongly related to timed up-and-go (TUG) test performance [18]. We have previously identified four distinct disability trajectories in the same cohort and the “disability-free” trajectory was characterised by 0–1 difficulties with ADLs over 5 years [13]. Therefore, the disability-free state in our present analysis was defined as having difficulty with none or one ADL.

### Mortality

Information on date of death was obtained from NHS Digital, UK. The time to death was calculated as the time between age at baseline (2006–2007) and time of death (censored at 29th August 2012). Mortality follow-up was conducted over a median of 5.5 (95% CI 3.4–6.2) years to match the end of data collection (last interview on the 28th August 2012).

### Anthropometry

Weight was measured using a digital scale (to the nearest 0.1 kg). In view of the difficulties measuring height in very old adults, height was estimated from two measurements of the right-arm demi-span (to the nearest 0.1 cm) and averaged [19]. Body mass index was calculated as weight (kg)/height^2^ (m). Body weight (BW) was adjusted to a desired BMI in older adults of 22–27 kg/m^2^ and calculated as described in Berner et al. [20]. Briefly, actual body weight was adjusted to the nearest (ideal) weight, putting the individual into an age-appropriate (≥ 71 years) healthy BMI range associated with a decreased risk of mortality [7]. Furthermore, most of the extra weight in overweight and obese people is fat tissue without the same requirements for amino acids than muscle tissue. In addition, extra protein is required to build muscle tissue in underweight individuals [20].

### Other variables

Participants were categorised into those who had spent up to 9 years, 10–11 years, or 12 or more years in full-time education. Disease count was created by scoring the most common seven chronic diseases as either present (1) or absent (0) (cardiac, respiratory and cerebrovascular disease, arthritis, hypertension, diabetes mellitus, and cancer in the past 5 years) [21]. Global cognition was assessed with the Standardised Mini-Mental State Examination (SMMSE) [22] and a score < 26 was defined as cognitive impairment.

### Statistical analysis

Normality was assessed with *Q*–*Q* plots. Non-Gaussian distributed variables are presented as medians and interquartile ranges (IQR); categorical data are presented as percentages (with corresponding sample size). Protein intake was expressed as g/kg aBW/d as a continuous variable and as an intake lower or, higher or equal than 0.8 and 1.0 g/kg aBW/d [7, 12]. To determine the contribution of protein intake to transition probabilities to and from a disability state and to death over 5 years, we fitted multi-state models with three states: disability-free (0–1 ADL), disability (2–17 ADLs), and death (absorbing state). We made the assumption that the transition between disability-free and death was through a disability state. The illustration of the illness-death model and the matrix of indicators for the allowed transitions are shown in Fig. S3. Multi-state models describe the movement of an individual between a number of finite states in a continuous time stochastic process under the Markov assumption that the next state is only influenced by the current state [23]. This movement is governed by transition probabilities which represent the instantaneous risk of moving from one state to another. States can be transient or absorbing if no transitions are possible from that state (i.e., death) [24].

We fitted four multi-state models with increasing complexity: model l included age, and protein intake (g/kg aBW/d) (continuous), or protein intake dichotomized to 0.8, 1, or 1.2 g/kg aBW/d cut-off; model 2 was further adjusted for sex and years spent in full-time education; model 3 was also adjusted for energy intake, cognition [Standardised Mini-Mental State Examination test (SMMSE)], and number of chronic diseases; and in model 4, a term for PA was added. These variables were selected based on their clinical and statistical relevance to a stable parsimonious model that also described the disability trajectories accurately [13]. Missing values (education: *n* = 4 and SMMSE score: *n* = 1) were imputed with the modal value (9 years of full-time education), or the mean (SMMSE score of 26.8). To determine the interaction between protein intake and PA, we fitted two models in subsets of participants with the same level of PA at baseline (i.e. medium and high PA) adjusted for the same confounders as previous models. The model for low PA did not converge as there were insufficient individuals who transitioned from disability-free to disability (*n* = 5).

A quasi-Newton method, the Broyden–Fletcher–Goldfarb–Shanno (BFGS) algorithm, was used to maximise the likelihood. The results from the multi-state models are presented as hazard ratios (HR) and 95% confidence intervals (CI). Expected time spent in each of the states was also extracted and presented. Multi-state models were fitted with the *msm* package [25] and plotted with *ggplot2* in R v3.2.2. Point estimates and confidence intervals were used to assess statistical and clinical significance.

## Results

At baseline, 37.5% (*n* = 269) of the participants were disability-free and 62.5% (*n* = 448) in a disability state. Women, less physically active individuals and those with lower score in the SMMSE had more disability at baseline (Table 1).

**Table 1:** Participant characteristics by disability states at baseline

	Disability-free (*n* = 269)	Disability (*n* = 448)	All (*n* = 717)	*p* value^a^
Women % (*n*)	48.3 (130)	66.5 (298)	59.7 (428)	< 0.001
Weight (kg)	63.3 (55.9–73.6)	63.2 (54.8–71.6)	63.2 (55.2–72.2)	0.321
BMI (kg/m^2^)	23.4 (21.3–26.7)	24.4 (21.7–27.4)	24.2 (21.5–27.2)	0.043
Full-time education % (*n*)				0.161
≤ 9 years	61.2 (164)	65.4 (291)	63.8 (458)	
10–11 years	23.1 (62)	23.8 (106)	23.7 (170)	
≥ 12 years	15.7 (42)	10.8 (48)	12.5 (90)	
Physical activity % (*n*)				< 0.001
Low	2.6 (7)	26.1 (117)	17.3 (124)	
Medium	26.9 (72)	56.3 (252)	45.3 (324)	
High	70.5 (189)	17.6 (79)	37.4 (268)	
Total energy (MJ/day)	7.0 (5.9–8.8)	6.7 (5.5–8.1)	6.8 (5.6–8.3)	0.016
Protein (g/day)	64.5 (50.9–81.4)	58.9 (48.3–73.3)	61.3 (49.3–75.7)	< 0.001
Protein (% en)	15.7 (13.4–18.3)	15.0 (13.1–17.3)	15.4 (13.2–17.8)	0.019
Protein (g/kg BW/day)	1.03 (0.79–1.30)	0.98 (0.76–1.20)	0.99 (0.78–1.24)	0.033
Protein (g/kg aBW/d)	1.00 (0.79–1.25)	0.95 (0.76–1.18)	0.97 (0.78–1.20)	0.035
< 0.8 g/kg aBW/d % (*n*)	26.4 (71)	28.3 (127)	27.6 (198)	0.556
< 1.0 g/kg aBW/d % (*n*)	51.3 (138)	56.9 (255)	54.8 (393)	0.119
Chronic disease count	2 (1–3)	2 (2–3)	2 (1–3)	< 0.001
Cognitive impairment % (*n*)	14.5 (39)	27.7 (124)	22.9 (165)	< 0.001

*aBW* adjusted body weight, *BMI* body mass index

^a^Chi square test for discrete or Mann–Whitney *U* test for continuous variables. Values are medians and interquartile ranges unless stated otherwise. Cognitive impairment was defined as having a Standardised Mini-Mental State Examination Score < 26

### Disability transitions

Disability states at baseline, 18, 36, and 60 months are shown in Table S1. The number of transitions at adjacent states over 5 years is shown in Table S2. There were 197 transitions from disability-free to disability, but most transitions were from disability to death (*n* = 457). At 85 years, participants were expected to spend 0.80 years (95% CI 0.63–1.04) disability-free and 2.78 years (95% CI 2.63–2.92) with disability over 5 years (extracted from model 4 from Table 2: one unit increase per g/kg aBW/d).

**Table 2:** Hazard ratios (HR) and 95% confidence intervals for the contribution of protein intake (continuous, 0.8 and 1.0 cut-off) to incident disability, recovery from disability, and transition from disability to death over 5 years

	Unit increase, g/kg aBW/day		≥ 0.8 g/kg aBW/day		≥ 1 g/kg aBW/day	
	HR	95% CI	HR	95% CI	HR	95% CI
Incident disability (*n* = 197)						
Model 1	0.58	0.39–0.87	0.62	0.46–0.84	0.60	0.46–0.78
Model 2	0.66	0.43–1.00	0.66	0.48–0.90	0.63	0.48–0.82
Model 3	0.45	0.25–0.81	0.53	0.36–0.80	0.56	0.40–0.78
Model 4	0.44	0.24–0.83	0.50	0.31–0.80	0.49	0.33–0.73
Recovery from disability (*n* = 46)						
Model 1	1.16	0.92–1.45	0.71	0.35–1.45	0.85	0.46–1.59
Model 2	0.88	0.34–2.26	0.72	0.35–1.47	0.84	0.45–1.59
Model 3	1.16	0.35–3.81	0.69	0.29–1.63	1.02	0.49–2.10
Model 4	0.96	0.29–3.19	0.62	0.25–1.55	0.84	0.38–1.84
Disability to death (*n* = 457)						
Model 1	1.01	0.77–1.34	0.86	0.71–1.04	1.00	0.84–1.19
Model 2	0.96	0.72–1.27	0.86	0.71–1.03	0.99	0.83–1.18
Model 3	1.28	0.90–1.83	0.95	0.77–1.18	1.07	0.88–1.30
Model 4	1.26	0.88–1.81	0.91	0.73–1.14	1.03	0.85–1.26

Protein intake < 0.8 or < 1.0 g/kg aBW/d were the reference categories. *N* are the number of transitions. Model l only included protein intake (g/kg aBW/d) or protein intake per 0.8 or 1 g/kg aBW/d cut-off and age; model 2 was further adjusted for sex and education; model 3 was also adjusted for energy intake, Standardised Mini-Mental State Examination Score and number of chronic diseases; and model 4 was further adjusted for physical activity

*aBW* adjusted body weight, *CI* confidence intervals, *HR* hazard ratio

### Protein intake and disability-free, disability, and death transitions

**Fig. 1:**
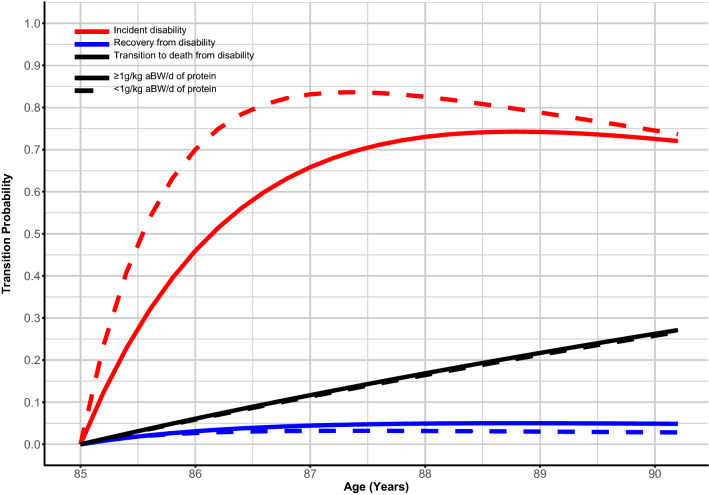
Probability of transition between disability states and to death by protein intake < and ≥ 1 g/kg aBW/d. *aBW* adjusted bodyweight. Red lines represent disability incidence, blue lines represent recovery from disability, and black lines represent death from disability. Full lines represent protein intake ≥ 1 g/kg aBW/d and dotted lines represent protein intake < 1 g/kg aBW/d. Models were adjusted for protein intake, age, sex, education, energy intake, Standardised Mini-Mental State Examination Score, number of chronic diseases, and physical activity

An increase in one unit of protein intake (g/kg aBW/d) (continuous) decreased the likelihood of incident disability in unadjusted models (HR 0.58, 95% CI 0.39–0.87) and in models adjusted for key covariates (e.g., model 4: HR 0.44, 95% CI 0.24–0.83) (Table 2). Significant reductions in incident disability were also found in individuals with protein intake ≥ 0.8 (HR 0.50, 95% CI 0.31–0.80) and ≥ 1 g/kg aBW/d (HR 0.49, 95% CI 0.33–0.73) (Table 2). Models with g/kg actual BW/d yielded similar results as with g/kg aBW/d (Table S3). Transition probabilities for recovery from disability, and death from disability were similar between participants with higher and lower protein intake (Fig. 1 and Table 2). Individuals with protein intake ≥ 0.8 (1.35 years, 95% CI 1.10–1.68) or ≥ 1 g/kg aBW/d (1.58 years, 95% CI 1.25–1.99) spent more years disability-free than those with protein intake < 0.8 (0.74 years, 95% CI 0.53–1.10) or < 1 g/kg aBW/d (0.86 years, 95% CI 0.66–1.11) (Table 3).

**Table 3:** Total time in years (95%CI) an 85 year old individual was expected to spend disability-free and with disability by protein intake cut-offs and low, medium, and high physical activity over 5 years

	< 0.8 g/kg aBW/d (*n* = 197)	≥ 0.8 g/kg aBW/d (*n* = 520)	< 1.0 g/kg aBW/d (*n* = 388)	≥ 1.0 g/kg aBW/d (*n* = 329)
Disability-free				
All (*n* = 269)	0.74 (0.53–1.10)	1.35 (1.10–1.68)	0.86 (0.66–1.11)	1.58 (1.25–1.99)
Low PA (*n* = 7)	0.27 (0.15–0.53)	0.53 (0.31–0.90)	0.31 (0.19–0.53)	0.62 (0.36–1.08)
Med PA (*n* = 72)	0.63 (0.43–0.92)	1.17 (0.91–1.51)	0.73 (0.54–0.99)	1.37 (1.03–1.82)
High PA (*n* = 189)	1.38 (1.02–1.90)	2.25 (1.90–2.62)	1.58 (1.28–2.00)	2.55 (2.13–2.98)
Disability				
All (*n* = 448)	3.69 (3.37–3.91)	3.22 (2.93–3.45)	3.63 (3.39–3.82)	3.01 (2.66–3.03)
Low PA (*n* = 117)	3.78 (3.49–3.99)	3.66 (3.35–3.89)	3.81 (3.55–3.99)	3.57 (3.20–3.83)
Med PA (*n* = 252)	3.74 (3.46–3.94)	3.35 (3.06–3.57)	3.70 (3.47–3.89)	3.16 (2.78–3.44)
High PA (*n* = 79)	3.26 (2.79–3.58)	2.50 (2.18–2.82)	3.10 (2.74–3.38)	2.24 (1.85–2.62)

Total time was extracted from the fully adjusted model in Table 2. Those models were adjusted for protein intake, age, sex, education, energy intake, Standardised Mini-Mental State Examination Score, number of chronic diseases, and physical activity. Participant numbers are from the baseline assessment. One participant (*n* = 1) did not have physical assessment

*aBW* adjusted body weight, *Med* medium, *PA* physical activity

### Protein intake × physical activity interaction and disability incidence

**Fig. 2:**
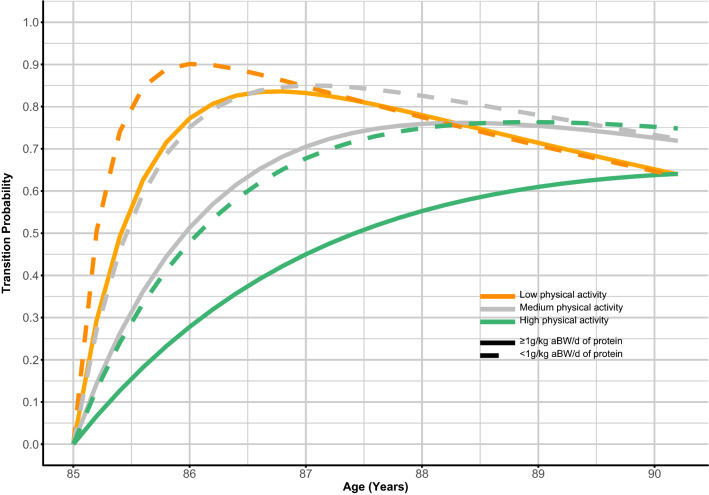
Probability of disability incidence by low, medium, and high physical activity levels and protein intake < and ≥ 1 g/kg aBW/d. *aBW* adjusted bodyweight. Orange lines represent low physical activity (PA), grey lines represent medium PA, and green lines represent high PA. Full lines represent protein intake ≥ 1 g/kg aBW/d and dotted lines represent protein intake < 1 g/kg aBW/d. Models were adjusted for protein intake, age, sex, education, energy intake, Standardised Mini-Mental State Examination Score, number of chronic diseases, and physical activity

The distribution of disability states by PA category at baseline, 18, 36 and 60 months is shown in Table S1. The number of transitions between disability states and death by PA category is shown in Table S2. There was no significant interaction in our sample between protein intake and PA in unadjusted and adjusted models (data not shown), but their benefit on disability incidence seemed to be additive, i.e., higher protein intake and higher PA were more protective than protein intake alone (Fig. 2, Tables 2, 3). There was also a lower probability of disability incidence within a given PA level when in combination with higher protein intake (Fig. 2). Physically active participants with higher protein intake were expected to spend more time disability-free than those within the same PA strata but low protein intake (Table 3). For example, an 85-year-old participant with high PA, but protein intake < 1 g/kg aBW/d was expected to spend 1.58 (1.28–2.00) years disability-free and 3.10 (2.74–3.38) years with disability, whilst a participant with the same level of PA and protein intake ≥ 1 g/kg aBW/d was expected to spend 2.55 (2.13–2.98) years disability-free and 2.24 (1.85–2.62) years with disability. Equally, participants with higher levels of PA and within the same protein intake strata spent more time disability-free and less time disabled (Table 3). Furthermore, to extract HRs for each PA category, we used subsets of participants with medium or high PA. Older adults with high PA, and higher protein intake (continuous, ≥ 0.8 and ≥ 1 g/kg aBW/d) were less likely to transition from disability-free to disability over 5 years than those within the same PA level but with lower protein intake (Table S4).

Higher protein intake in participants with medium PA also showed the same trend, although results were not as consistent as with high physical activity (Table S4).

## Discussion

### Main findings

Community-dwelling very old adults with higher protein intakes were less likely to transition from disability-free to disability over 5 years from age 85 to 90 years. As a result, transition from disability-free to disability happened at a later age in those with higher than in those with lower protein intake. Participants with higher PA and higher protein intake were less likely to transition to disability than those within the same PA level but with lower protein intake.

### Disability transitions

At baseline, almost 2/3 of the participants (62.5%) were already in a disability state. This was reflected in the reduced number of incident disability transitions (*n* = 197) and reduced power to observe an effect of protein intake on disability progression. It is noteworthy that although not associated with protein intake, nearly 50 participants recovered from disability over the 5 years in our study. This draws attention to the fact that development of disability does not lead, inevitably, to death, but that recovery is possible, even at advanced age [26].

### Protein intake and disability incidence

In this study, functional decline, from disability-free to disability, was less likely in very old adults with higher protein intake (measured at baseline) using multi-state models and adjusting for key confounders. Previously, we showed that very old adults with higher protein intake, especially ≥ 1.0 g/kg aBW/d, were more likely to have a disability-free trajectory over 5 years [13]. This is in line with the other studies reporting that older adults living in the community with higher protein intake, especially ≥ 0.8–1.0 g/kg BW/d, had lower prevalence and incidence of disabilities [27–30]. These studies included younger participants (aged 67–79), assessed dietary intake with food frequency questionnaires (FFQ) or 24 h recalls, followed participants for 24 days–13 years, used different ways of identifying functional limitations, and did not account for non-random dropout. For example, Beasley et al. included 110,000 + post-menopausal women aged 50–79 from the Women’s Health Initiative (WHI) study (WHI observational study and clinical trials), and found that those with higher protein intake at baseline (assessed by a FFQ and calibrated for BMI, age, ethnicity, and smoking status) had slower rate of functional decline (e.g., walking, bathing, dressing, and climbing stairs) over 11.5 ± 3.1 years [28]. Sufficient dietary energy is required for dietary protein to optimally stimulate muscle protein synthesis and mitigate the losses of muscle mass and strength [31]. Participants with disability had slightly lower energy intake than those disability-free at baseline and higher protein intake in the presence of insufficient energy intake (energy deficit) might not be as effective. We have accounted for this by adjusting our models for energy intake but using energy-adjusted protein intake (residual method) as the exposure would be interesting.

### Protein intake and recovery from disability

We did not find meaningful differences between participants with higher and lower protein intake and their probability of recovering from disability. This might be a reflection of the low number of recovery transitions and hence reduced statistical power to detect significant differences. Another possible explanation is that the assignment of disability in the previous wave (prior to the recovery) was due to transient events and not chronic disability where dietary protein might have a protective effect through muscle mass and strength. To the best of our knowledge, there are no studies that evaluated protein intake and disability recovery in the very old.

### Protein intake and transition to death from disability

We also did not find differences in the transition probability from disability to death in participants with low and high protein intake. Although observational studies have found an association between protein intake and mortality [32], our rationale for including transitions from disability to death was mainly statistical and not biological. Attrition is high in this age group and in our cohort [33], and failure to account for mortality (non-random attrition) would likely result in biased estimates [34]. However, this result might suggest that adequate protein intake is more important during the early stages of disability progression and not when the disability process is well established.

### Protein intake × physical activity interaction and disability incidence

Overall, very old adults with high PA and high protein intake transitioned at a later age from disability-free to disability compared with those within the same PA level but with lower protein intake. However, this effect could not be investigated adequately within the low PA participants because of insufficient numbers of individuals who transitioned to disability and had low PA levels. This limitation emphasizes how the level of PA can be a cause and a consequence of the disability process and how it is not easy to disentangle both. We found no evidence of an interaction between PA and protein intake (possibly due to low power), but the benefits of higher protein and higher PA on disability incidence appear to be additive (e.g., higher protein intake and higher PA were more protective compared with protein intake alone).

Expert groups, such as PROT-AGE, have suggested that there might be a synergistic protective effect of protein and physical activity on age-related muscle strength and muscle mass loss [6]. This would be anticipated if high PA and adequate protein intake together were necessary to stimulate the rate of muscle synthesis optimally. Such a combination would be expected to decrease the rate of loss of muscle strength and mass and to slow the rate of functional decline in older adults for ADLs involving muscle strength. A meta-analysis of nine randomised-controlled trails (RCTs) including 462 older adults (61–79 years) found that protein supplementation in combination with resistance training for 6 weeks or longer resulted in increased fat-free mass but not in greater muscle strength or muscle mass compared with the control group with no protein supplementation or a low protein diet combined with the same level of exercise [35]. A later systematic review of 15 RCTs (with only one study in common between both reviews) in older adults (mean age of 77) on the additive effect of protein supplementation and progressive resistance training (2–5 times/week for 7 weeks–1 year) found that improvements in functional ability (gait speed, timed up-and-go, balance test, chair rises, and stair climb), muscle strength, muscle mass, and body composition were not different between resistance training alone or in combination with protein [36]. However, the authors report that the findings suggested a synergistic effect of resistance training and protein supplementation in frail older adults with lower protein intake [36]. We have reported that very old adults with higher levels of PA and protein intake ≥ 1 g/kg aBW/d had higher grip strength (GS) at baseline and that GS declined slower than in those with low PA [12]. This effect was not seen in participants with high PA but low protein intake or low PA but high protein intake [12]. This suggests that an adequate protein intake and higher PA level are necessary. More research is needed to clarify the possible additive effects of high protein intake (≥ 1 g/kg aBW/d) and high PA in delaying incident disability in very old adults.

### Strengths and weaknesses

Self-reported difficulty in performing ADLs was collected 18 months apart between each data collection wave, and it is possible that unobserved transitions from and to disability occurred between each wave. There were few individuals with low levels of PA who transitioned or recovered from disability, and therefore, results concerning this have to be taken carefully. This is likely to be similar in other cohorts of advanced age but nonetheless an important limitation. Protein intake was measured at baseline only and, therefore, effects were assumed to be stable over 5 years; protein intake assessment at other data collection waves may have yielded different results. The large range of difficulties performing ADLs collected and the use of multi-state models to understand the contribution of protein to transitions between disability states and to death are major strengths of this study.

## Conclusion

Higher protein intake alone or in combination with higher PA may delay the incidence of disability in very old adults. More research is needed to clarify the possible additive effect of higher protein intake and higher PA in delaying incident disability in very old adults.

## Electronic supplementary material

Below is the link to the electronic supplementary material.

Supplementary material 1 (DOCX 121 kb)
