# Supplementary material for: Contribution of protein intake and its interaction with physical activity to transitions between disability states and to death in very old adults: the Newcastle 85+ Study
Source: Eur J Nutr. 2019 Jul 10;59(5):1909–18. doi: 10.1007/s00394-019-02041-1 (PMC7351810; doi:10.1007/s00394-019-02041-1)

**European Journal of Nutrition**

***Contribution of protein intake and its interaction with physical activity to transitions between disability states and to death in the very old adults: The Newcastle 85+ Study***

Nuno Mendonça^1-3*^, Andrew Kingston^1,3^, Antoneta Granic^1,4,5^, Tom R. Hill^2,6^, John C. Mathers^1,2,6^, Carol Jagger^1,3^

^1^ Newcastle University Institute for Ageing, Newcastle University, NE2 4AX, UK.

^2^ Human Nutrition Research Centre, Newcastle University, NE2 4HH, UK.

^3^ Institute of Health and Society, Newcastle University, NE4 5PL, UK.

^4^ AGE Research Group, Institute of Neuroscience, Newcastle University, NE2 4HH, UK.

^5^ NIHR Newcastle Biomedical Research Centre in Ageing and Chronic Disease, Newcastle University and Newcastle upon Tyne NHS Foundation Trust, NE4 5PL, UK.

^6^ Institute of Cellular Medicine, Newcastle University, NE2 4HH, UK.

*****Corresponding author: Nuno Mendonça, email: [nuno.mendonca@newcastle.ac.uk](mailto:nuno.mendonca@newcastle.ac.uk), ORCID: 0000-0001-7589-9901

**Supplementary Material**

**Table S1.** Disability states distribution in Newcastle 85+ Study by physical activity and follow-up.

**Table S2.** Number of transitions between disability-free, disability and to death for all participants or by low, medium and high physical activity over 5 years.

**Table S3.** Hazard ratios (HR) and 95% confidence intervals for the contribution of protein intake in g/kg actual bodyweight/d (continuous, 0.8 and 1.0 cut-off) to incident disability, recovery from disability and transition from disability to death over 5 years.

**Table S4.** Hazard ratios (HR) and 95% confidence intervals for the contribution of protein intake to transitions between disability states and to death per medium and high physical activity strata over 5 years.

**Fig S1** Flowchart of recruitment and cohort retention profile of the Newcastle 85+ Study according to the sample used

**Fig S2** The 17 basic (BADL) and instrumental activities of daily living (IADL) and mobility activities used to derive the disability score

**Fig S3** Disability-death multi-state model and allowed transitions

**Table S1.** Disability states distribution in Newcastle 85+ Study by physical activity and follow-up.

|  | **Low PA** | **Medium PA** | **High PA** | **All** |
| --- | --- | --- | --- | --- |
| **Baseline %(n)** | **n=124** | **n=324** | **n=268** | **n=717** |
| Disability-free | 5.6 (7) | 22.2 (72) | 70.5 (189) | 37.5 (269) |
| Disability | 94.4 (117) | 77.8 (252) | 29.5 (79) | 62.5 (448) |
| **18 Months %(n)** | **n=74** | **n=273** | **n=232** | **n=580** |
| Disability-free | - | 11.7 (32) | 38.4 (89) | 21.0 (122) |
| Disability | 100 (74) | 88.3 (241) | 61.6 (143) | 79.0 (458) |
| **36 Months %(n)** | **n=44** | **n=213** | **n=194** | **n=452** |
| Disability-free | - | 5.2 (11) | 30.4 (59) | 15.5 (70) |
| Disability | 100 (44) | 94.8 (202) | 69.6 (135) | 84.5 (382) |
| **60 Months %(n)** | **n=26** | **n=152** | **n=148** | **n=327** |
| Disability-free | 3.8 (1) | 2.0 (3) | 29.7 (44) | 14.7 (48) |
| Disability | 96.2 (25) | 98 (149) | 70.3 (104) | 85.3 (279) |

PA, physical activity. One participant (n=1) does not have physical activity at baseline and follow-up.

**Table S2.** Number of transitions between disability-free, disability and to death for all participants or by low, medium and high physical activity over 5 years.

|  |  | **To** | Disability-free | Disability | Death |
| --- | --- | --- | --- | --- | --- |
|  | **From** |  |  |  |  |
| All | Disability-free |  | 194 | 197 | 69 |
|  | Disability |  | 46 | 914 | 457 |
| Low PA | Disability-free |  | 0 | 5 | 2 |
|  | Disability |  | 1 | 136 | 113 |
| Medium PA | Disability-free |  | 26 | 72 | 16 |
|  | Disability |  | 21 | 519 | 226 |
| High PA | Disability-free |  | 168 | 120 | 51 |
|  | Disability |  | 24 | 259 | 118 |

PA, physical activity.

**Table S3.** Hazard ratios (HR) and 95% confidence intervals for the contribution of protein intake in g/kg actual bodyweight/d (continuous, 0.8 and 1.0 cut-off) to incident disability, recovery from disability and transition from disability to death over 5 years.

|  | **unit increase**  **g/kg BW/day** | | **≥0.8 g/kg BW/day** | | **≥1 g/kg BW/day** | |
| --- | --- | --- | --- | --- | --- | --- |
|  | HR | 95% CI | HR | 95% CI | HR | 95% CI |
|  | **Incident Disability** (n=197) | | | | | |
| Model 1 | 0.62 | 0.44-0.88 | 0.64 | 0.48-0.87 | 0.64 | 0.50-0.84 |
| Model 2 | 0.68 | 0.47-0.98 | 0.67 | 0.49-0.91 | 0.67 | 0.51-0.88 |
| Model 3 | 0.51 | 0.31-0.84 | 0.57 | 0.39-0.84 | 0.58 | 0.42-0.81 |
| Model 4 | 0.52 | 0.31-0.87 | 0.53 | 0.34-0.83 | 0.55 | 0.37-0.81 |
|  | **Recovery from Disability** (n=46) | | | | | |
| Model 1 | 0.82 | 0.35-1.94 | 0.73 | 0.37-1.46 | 0.73 | 0.39-1.35 |
| Model 2 | 0.84 | 0.36-1.99 | 0.74 | 0.37-1.48 | 0.72 | 0.39-1.36 |
| Model 3 | 0.90 | 0.32-2.55 | 0.66 | 0.29-1.50 | 0.78 | 0.38-1.59 |
| Model 4 | 0.85 | 0.31-2.37 | 0.69 | 0.29-1.64 | 0.67 | 0.31-1.45 |
|  | **Disability to Death** (n=457) | | | | | |
| Model 1 | 1.08 | 0.85-1.38 | 0.94 | 0.78-1.14 | 0.99 | 0.83-1.18 |
| Model 2 | 1.05 | 0.82-1.33 | 0.94 | 0.78-1.4 | 1.01 | 0.85-1.20 |
| Model 3 | 1.38 | 1.03-1.85 | 1.06 | 0.86-1.31 | 1.10 | 0.91-1.34 |
| Model 4 | 1.35 | 1.00-1.82 | 1.01 | 0.82-1.25 | 1.08 | 0.89-1.31 |

BW, body weight; CI, confidence intervals; HR, hazard ratio. Protein intake <0.8 or < 1.0 g/kg BW/d was the reference category. N are the number of transitions. Model l only included protein intake (g/kg BW/d) or protein intake per 0.8 or 1 g/kg BW/d cut-off and age; Model 2 was further adjusted for sex and education; Model 3 was also adjusted for energy intake, Standardised Mini-Mental State Examination Score and number of chronic diseases; and Model 4 was further adjusted for physical activity.

**Table S4.** Contribution of protein intake to transitions between disability states and to death per medium and high physical activity strata over 5 years.

|  | **Medium Physical activity** | | | | | | **High Physical activity** | | | | | |
| --- | --- | --- | --- | --- | --- | --- | --- | --- | --- | --- | --- | --- |
|  | **unit increase**  **g/kg aBW/day** | | **≥0.8 g/kg aBW/day** | | **≥1 g/kg aBW/day** | | **unit increase**  **g/kg aBW/day** | | **≥0.8 g/kg aBW/day** | | **≥1 g/kg aBW/day** | |
|  | HR | 95% CI | HR | 95% CI | HR | 95% CI | HR | 95% CI | HR | 95% CI | HR | 95% CI |
|  | **Incident Disability** (n=72) | | | | | | **Incident Disability** (n=120) | | | | | |
| Model 1 | 1.16 | 0.40-3.38 | 0.93 | 0.52-1.65 | 0.70 | 0.40-1.22 | 0.57 | 0.35-0.94 | 0.49 | 0.30-0.81 | 0.53 | 0.37-0.77 |
| Model 2 | 1.17 | 0.40-3.45 | 0.92 | 0.51-1.66 | 0.70 | 0.40-1.23 | 0.64 | 0.38-1.07 | 0.48 | 0.28-0.83 | 0.54 | 0.37-0.80 |
| Model 3 | 0.56 | 0.13-2.31 | 0.48 | 0.20-1.14 | 0.39 | 0.17-0.91 | 0.43 | 0.21-0.89 | 0.27 | 0.03-2.63 | 0.45 | 0.28-0.72 |
|  | **Recovery from Disability** (n=21) | | | | | | **Recovery from Disability** (n=24) | | | | | |
| Model 1 | 1.97 | 0.40-9.73 | 1.89 | 0.56-6.44 | 1.48 | 0.55-4.00 | 0.45 | 0.12-1.74 | 0.30 | 0.11-0.86 | 0.48 | 0.18-1.24 |
| Model 2 | 1.84 | 0.34-9.90 | 1.74 | 0.52-5.83 | 1.44 | 0.53-3.90 | 0.46 | 0.12-1.74 | 0.28 | 0.10-0.82 | 0.45 | 0.17-1.19 |
| Model 3 | 2.70 | 0.32-22.42 | 1.88 | 0.41-8.58 | 1.62 | 0.45-5.85 | 0.44 | 0.08-2.35 | 0.16 | 0.01-4.12 | 0.43 | 0.14-1.30 |
|  | **Disability to Death** (n=226) | | | | | | **Disability to Death** (n=118) | | | | | |
| Model 1 | 0.94 | 0.59-1.50 | 0.80 | 0.61-1.04 | 0.87 | 0.67-1.13 | 1.21 | 0.80-1.82 | 1.07 | 0.74-1.56 | 1.38 | 1.02-1.89 |
| Model 2 | 0.84 | 0.53-1.33 | 0.81 | 0.62-1.06 | 0.85 | 0.66-1.10 | 1.15 | 0.75-1.77 | 0.98 | 0.67-1.44 | 1.35 | 0.98-1.85 |
| Model 3 | 1.26 | 0.72-2.19 | 0.97 | 0.70-1.33 | 0.97 | 0.73-1.29 | 1.31 | 0.74-2.34 | 0.98 | 0.65-1.50 | 1.48 | 1.04-2.11 |

aBW, adjusted body weight; CI, confidence intervals; HR, hazard ratio. Protein intake <0.8 and <1.0 g/kg aBW/d are the reference categories. Model l only included protein intake (g/kg aBW/d) or protein intake per 0.8 or 1 g/kg aBW/d cut-off and age; Model 2 was further adjusted for sex and education; and Model 3 was also adjusted for energy intake, Standardised Mini-Mental State Examination Score and number of chronic diseases. Hazard ratios for low physical activity could not be calculated because of insufficient transitions. N are the number of transitions in each strata.

**Fig S1** Flowchart of recruitment and cohort retention profile of the Newcastle 85+ Study according to the sample used


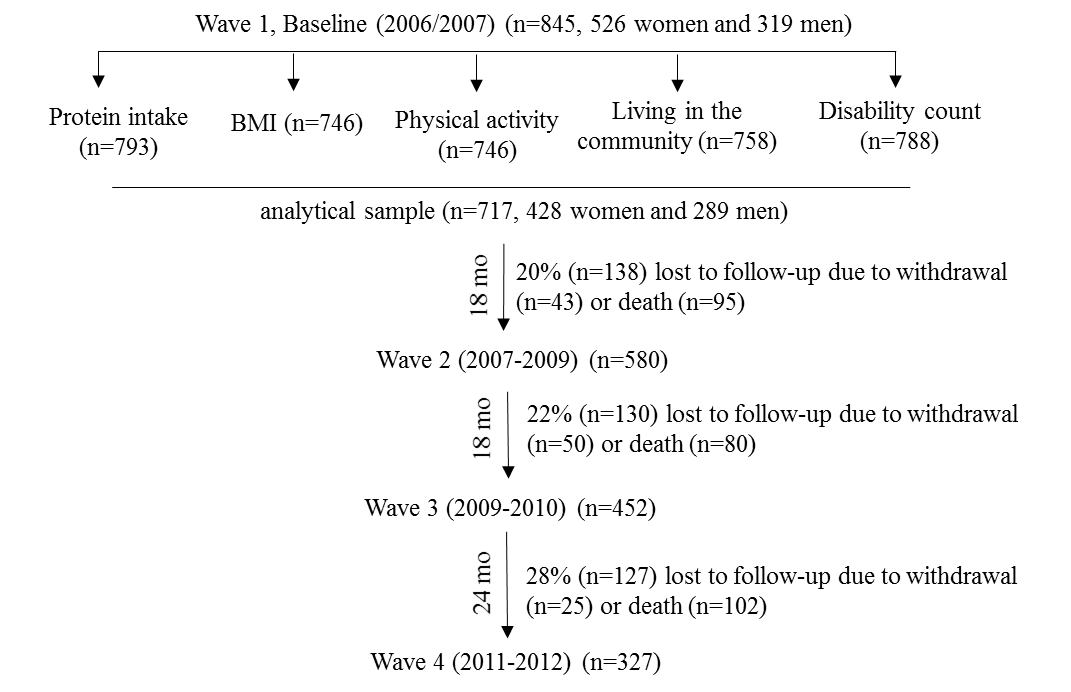


BMI, body mass index; GP, general practitioner; mo, months.

**Fig S2** The 17 basic (BADL) and instrumental activities of daily living (IADL) and mobility items used to derive the disability score

| BADL | Are you able to get in and out of bed? |
| --- | --- |
|  | Are you able to get and out of a chair? |
|  | Are you able to get on and off the toilet? |
|  | Are you able to dress and undress yourself? |
|  | Are you able to wash your face and hands? |
|  | Are you able to wash yourself all over? |
|  | Are you able to cut your own toenails? |
|  | Are you able to feed yourself? |
| IADL | Are you able to cook a hot meal? |
|  | Are you able to shop for your groceries? |
|  | Are you able to do light housework? |
|  | Are you able to do heavy housework? |
|  | Are you able to manage money? |
|  | Are you able to manage your medications? |
| MOBILITY | Are you able to up and down stairs/steps? |
|  | Are you able to get around the house? |
|  | Are you able to walk 400 yards (366 m) |

**Fig S3** Disability-death multi-state model and allowed transitions


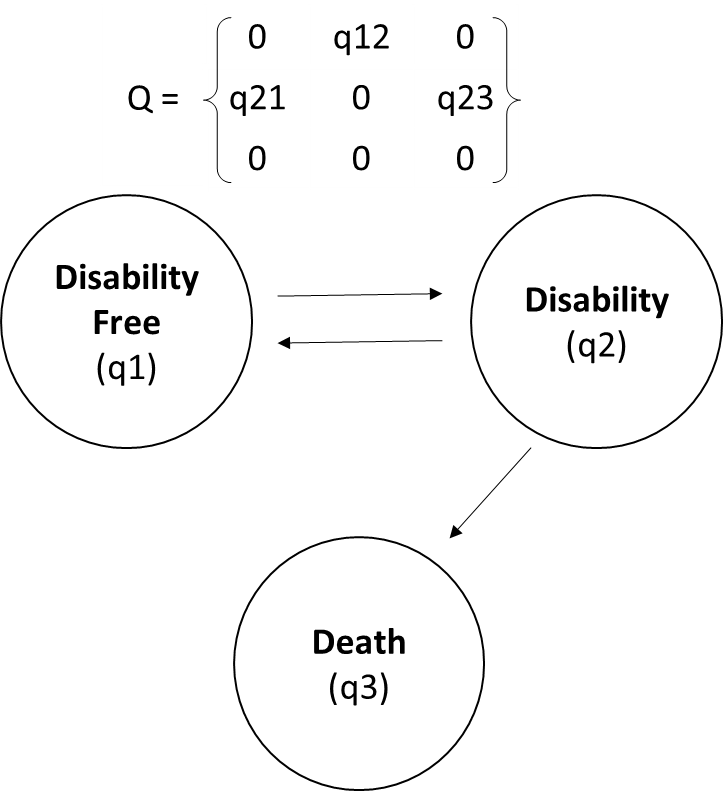

Supplement: Supplementary file 1 — Supplementary material 1 (DOCX 121 kb) [file 394_2019_2041_MOESM1_ESM.docx]
